# Thalidomide in combination with augmentin (amoxicillin with clavulanic acid) protects BALB/c mice suffering from *Klebsiella pneumoniae *B5055-induced sepsis

**DOI:** 10.1186/cc10397

**Published:** 2011-10-27

**Authors:** V Kumar, S Chhibber

**Affiliations:** 1 Department of Microbiology, Panjab Univeristy, Chandigarh, India

## Introduction

Despite extensive research in the field of sepsis pathogenesis and its management, mortality associated with sepsis in hospitals remains very high. For example, more than 18 million people are affected by sepsis worldwide and have an expected 1% increase annually in ICUs. Sepsis is the outcome of a deregulated immune system occurring during systemic bacterial (that is, Gram-negative or Gram-positive) infection. So modulating the immune system by an immunomodulatory approach may prove beneficial to sepsis patients. In the present study, we evaluated the protective immunomodulatory effect of thalidomide alone or with augmentin in *Klebsiella pneumoniae *B5055-induced sepsis in BALB/c mice.

## Methods

The mouse model of sepsis was developed by placing *K. pneumoniae *B5055 entrapped in fibrin and thrombin clots in the peritoneal cavity of mice. The septic mice were treated with thalidomide alone (30 mg/kg/day p.o.), with augmentin alone (20 μg/ml i.p.) and with their combination. The bacterial load in blood was estimated by blood culture on MacConkey's agar plates along with measuring the other systemic inflammatory parameters. For example, lipid peroxidation was measured in terms of malondialdehyde (MDA) and nitric oxide (NO) levels in serum by biochemical methods. Levels of proinflammatory cytokines (that is, TNFα and IL-1α) and anti-inflammatory cytokine (that is, IL-10) levels in serum were measured by ELISA.

## Results

The thalidomide-alone-treated mice showed 75% survival whereas 60% of the augmentin-alone-treated group survived. However, their combination (thalidomide + augmentin) treatment provided 100% survival. Treatment with thalidomide alone significantly (*P *< 0.05) decreased TNFα, IL-1α, NO and MDA levels in the serum without significantly (*P *< 0.05) decreasing the bacterial count in blood. However, levels of IL-10 in serum were found to be significantly (*P *< 0.05) elevated upon thalidomide treatment. Augmentin alone only decreased the bacterial load in blood significantly (*P *< 0.05), while no significant decrease was observed on inflammatory mediators studied. However, a combination thalidomide with augmentin significantly (*P *< 0.05) decreased both the bacterial count as well as inflammatory mediators (that is, TNFα, IL-1α, NO and MDA) and provided 100% protection to animals.

## Conclusion

Thalidomide can be used as an immunomodulatory agent along with antibiotics for sepsis management.

